# Karyomegalic interstitial nephritis and DNA damage-induced polyploidy in Fan1 nuclease-defective knock-in mice

**DOI:** 10.1101/gad.276287.115

**Published:** 2016-03-15

**Authors:** Christophe Lachaud, Meghan Slean, Francesco Marchesi, Claire Lock, Edward Odell, Dennis Castor, Rachel Toth, John Rouse

**Affiliations:** 1 MRC Protein Phosphorylation and Ubiquitylation Unit, College of Life Sciences, Sir James Black Centre, University of Dundee, Dundee DD1 5EH, United Kingdom;; 2 School of Veterinary Medicine, College of Medical, Veterinary, and Life Sciences, University of Glasgow, Glasgow G61 1QH, United Kingdom;; 3 Department of Head and Neck Pathology, Guy's Hospital, London SE1 9RT, United Kingdom

**Keywords:** FAN1, FANCD2, ICL, Fanconi anemia, KIN, karyomegaly

## Abstract

The Fan1 endonuclease is required for repair of DNA interstrand cross-links (ICLs). Mutations in human Fan1 cause karyomegalic interstitial nephritis (KIN), but it is unclear whether defective ICL repair is responsible or whether Fan1 nuclease activity is relevant. We show that Fan1 nuclease-defective (*Fan1*^nd/nd^) mice develop a mild form of KIN. The karyomegalic nuclei from *Fan1*^nd/nd^ kidneys are polyploid, and fibroblasts from *Fan1*^nd/nd^ mice become polyploid upon ICL induction, suggesting that defective ICL repair causes karyomegaly. Thus, Fan1 nuclease activity promotes ICL repair in a manner that controls ploidy, a role that we show is not shared by the Fanconi anemia pathway or the Slx4–Slx1 nuclease also involved in ICL repair.

## Abstract (precis)

In this study, Lachaud et al. investigated the cause of karyomegalic interstitial nephritis (KIN), a form of chronic kidney disease characterized by karyomegaly. They demonstrate that mice lacking Fan1 nuclease activity recapitulate the symptoms of KIN, providing new insights into how Fan1 nuclease activity contributes to the KIN phenotype.

DNA interstrand cross-links (ICLs) are toxic lesions that block the progression of replisomes. Defects in ICL repair are associated with Fanconi anemia (FA), a rare recessive disorder typified by developmental abnormalities, bone marrow failure, and increased incidence of cancers (Auerbach 2009; Kim and D'Andrea 2012). There are currently 19 genes known to cause FA when mutated, and studying the products of the FA genes has shed important insights into how ICLs are repaired. A central event in ICL repair is the monoubiquitylation of Fancd2 catalyzed by the multisubunit FA core complex, which acts as an E3 ubiquitin ligase in concert with Ube2t (Akkari et al. 2000; Garcia-Higuera et al. 2001; Alpi et al. 2008). Preventing Fancd2 ubiquitylation (K561) causes defective ICL repair (Garcia-Higuera et al. 2001; Knipscheer et al. 2009; Deans and West 2011).

Fan1 is a 5′ flap endonuclease recruited to sites where replisomes stall—for example, at ICLs—in a manner that requires Fancd2 ubiquitylation (Kratz et al. 2010; Liu et al. 2010; MacKay et al. 2010; Smogorzewska et al. 2010). The nuclease activities of Fan1 are mediated by a C-terminal “VRR_nuc” domain (Iyer et al. 2006), which is conserved in all orthologs as far back as yeasts. Fan1 also has a SAP-type DNA-binding domain and a UBZ4-type ubiquitin-binding domain, which interacts with ubiquityl-Fancd2, thereby recruiting Fan1 to sites of replisome stalling. Fan1 is required for efficient ICL repair, but, surprisingly, this does not require interaction with ubiquityl-Fancd2 (Lachaud et al. 2016). Similarly, genetic analyses in worms and human cells showed that Fan1 and Fancd2 mutations are not epistatic with respect to hypersensitivity to ICL-inducing drugs, suggesting that the two genes are not equivalent in function when it comes to ICL repair (Yoshikiyo et al. 2010; Zhou et al. 2012).

Consistent with the idea that the role of Fan1 in ICL repair is different from the FA pathway, biallelic mutations in *Fan1* do not cause FA. Instead, they cause a form of chronic kidney disease referred to as karyomegalic interstitial nephritis (KIN) (Zhou et al. 2012). KIN is characterized by dilation and fibrosis of renal tubules with immune cell invasion of interstitial spaces but differs from most other forms of chronic kidney disease in that it is accompanied by pronounced karyomegaly in the renal tubular epithelium (Godin et al. 1996). The cause of the karyomegaly seen in KIN is not understood, and it is not clear whether this or any of the other characteristics of KIN in patients with Fan1 mutations is caused by defective ICL repair. Furthermore, it is not clear whether the nuclease activity of Fan1 is required to prevent KIN, an issue that we address in this study.

## Results and Discussion

### Fan1 nuclease-defective mice (*Fan1*^nd/nd^) exhibit hallmarks of KIN

**Figure 1.**
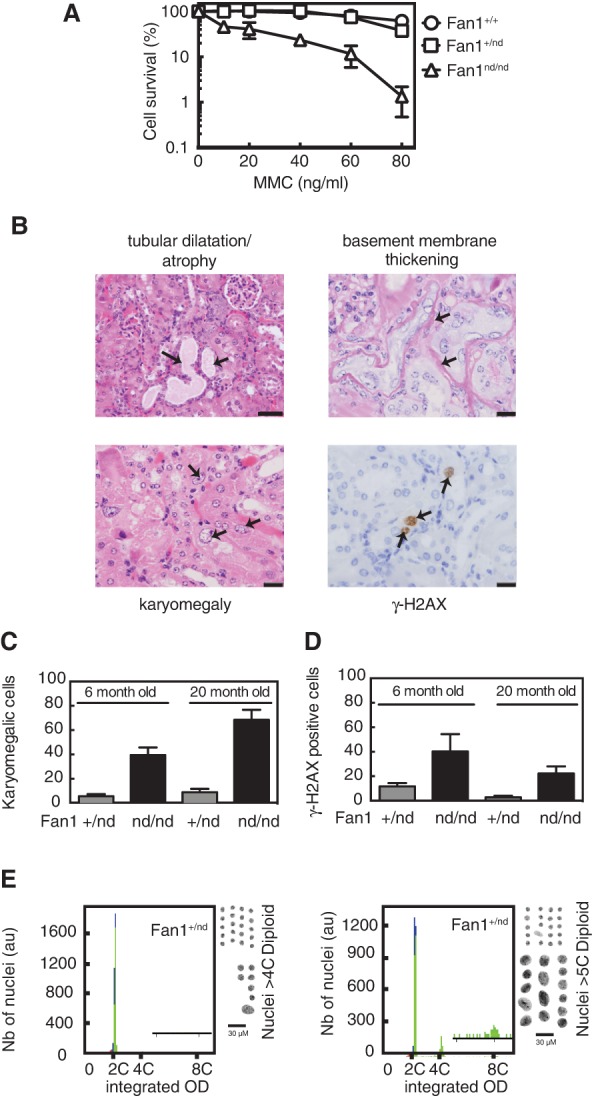
*Fan1*^nd/nd^ mice show KIN, karyomegaly, and altered ploidy. (*A*) Clonogenic survival analysis of MEFs exposed to MMC. For each genotype, cell viability of untreated cells is defined as 100%. Data are represented as mean ± SD. *n* = 3. (*B*) Representative images of hematoxylin and eosin (HE)-stained kidney sections from *Fan1*^nd/nd^ mice at 20 mo of age demonstrating atrophy of renal tubular epithelium, basement membrane thickening, and karyomegaly. A representative image of a kidney section stained with γ-H2AX antibodies is also shown. (*C*,*D*) The incidence of karyomegaly in 1000 cells per mouse was quantitated in three mice per genotype at 6 mo and 20 mo of age. Karyomegaly was counted when the size of a nucleus exceeded three times the size of other nuclei within the same field. Data are represented as mean ± SD. (*E*) DNA ploidy histograms (*left* panels) and separated nuclei (*right* panels) for kidneys from *Fan1*^+/nd^ and *Fan1*^nd/nd^ mice.

To test the importance of the nuclease activity of Fan1 for ICL repair, we introduced a *Fan1*-inactivating mutation in the mouse genome to make knock-in mice. Specifically, we introduced a mutation in the codon for K975 in the VRR_nuc domain at the C terminus of Fan1 (Lachaud et al. 2016). This mutation also had the consequence of inhibiting splicing of exon 13 with exon 14 (the last *Fan1* exon), resulting in intron retention and deletion of the last 41 amino acids of Fan1, including several residues essential for catalysis (Lachaud et al. 2016). Consequently, the truncated form of Fan1 encoded by the *Fan1-nd* allele is devoid of nuclease activity (Supplemental Fig. S1). Mouse embryonic fibroblasts (MEFs) isolated from homozygous *Fan1*^nd/nd^ mice are profoundly hypersensitive to ICL-inducing genotoxins, but MEFs from *Fan1*^+/nd^ mice are not, indicating that the nuclease-dead Fan1 mutant does not act in a dominant-negative manner (Fig. 1A). MEFs from *Fan1*^nd/nd^ mice also show pronounced G_2_ arrest after exposure to mitomycin C (MMC) or diepoxybutane (DEB) (Supplemental Fig. S2A). These data show that Fan1 nuclease activity is important for ICL repair. MEFs from *Fan1*^nd/nd^ mice also show an increase in the frequency of chromosome abnormalities after MMC or DEB (Supplemental Fig. S2B), a defect that was recently shown to reflect a role in the processing of stalled forks independent of ICL repair (Lachaud et al. 2016).

*Fan1*^+/nd^ and *Fan1*^nd/nd^ mice were born at the expected Mendelian frequencies (Supplemental Table S1) without overt morphological, developmental, or hematological defects, and adult mice were fertile (data not shown). However, we observed signs of KIN in *Fan1*^nd/nd^ mice, and the most notable feature was prominent karyomegaly in the renal tubular epithelium, coincident with positive γ-H2AX staining (Fig. 1B,C). Karyomegaly was evident at 6 mo of age and worse by 20 mo (Fig. 1B,C; Supplemental Table S2). Renal histopathology also revealed an increased incidence of a spectrum of tubular changes, particularly in older mice, including dilation, degeneration, and atrophy, along with increased infiltration of inflammatory cells in interstitial spaces (Fig. 1B; Supplemental Table S2). Furthermore, tubules in the older mice were frequently characterized by thickening of the basement membranes (Fig. 1B; Supplemental Table S2).

To understand the nature of the karyomegaly in *Fan1*^nd/nd^ mice, we measured the DNA content of nuclei isolated from kidney sections. Nuclei from Fan1^+/nd^ mice were diploid with a single peak at 2n, minimal G_2_ population (4n), and no detectable S-phase population (Fig. 1E). On the other hand, nuclei from *Fan1*^nd/nd^ mice showed an increased population with 4n DNA content and a minority of nuclei with 8n content, suggestive of whole-genome or near-whole-genome doublings without division in a subset of cells (Fig. 1E; Supplemental Fig. S3). Taken together, these data show that Fan1 nuclease activity is critical for preventing alterations in ploidy in the renal tubular epithelium and preventing symptoms of KIN.

### Polyploidy in MEFs from *Fan1*^nd/nd^ mice upon ICL induction

**Figure 2.**
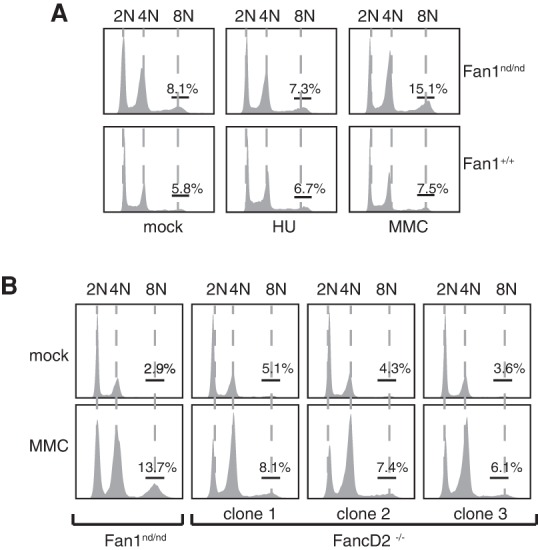
Polyploidy in MEFs from Fan1^nd/nd^ mice upon ICL induction. Primary MEFs of the genotypes indicated were exposed to 50 ng/mL MMC for 24 h or 0.5 mM HU for 18 h before cells were fixed, stained with propidium iodide, and subjected to FACS analysis.

Karyomegaly in the kidneys of *Fan1*^nd/nd^ mice may be a consequence of unrepaired ICLs that arise in kidneys through continuous filtration of genotoxic waste compounds. We reasoned that embryonic fibroblasts from *Fan1*^nd/nd^ mice would not have sustained such a high level of genotoxic insult but may become polyploid upon ICL induction. As shown in Figure 2A, exposure of primary MEFs from Fan1^nd/nd^ mice, but not from control *Fan1*^+/+^ mice, led to a modest but significant increase in the proportion of cells with 8n DNA content, reminiscent of the ploidy changes observed in kidneys from *Fan1*^nd/nd^ mice (Fig. 1E; Supplemental Fig. S3). These changes in ploidy after MMC were consistently observed in primary *Fan1*^nd/nd^ MEFs but not once the cells were immortalized (data not shown). Treatment of primary *Fan1*^nd/nd^ MEFs with hydroxyurea, which stalls replication forks by depleting deoxyribonucleotides, did not cause polyploidy (Fig. 2A), indicating that polyploidy is a consequence of unrepaired ICLs as opposed to defective processing of stalled replication forks. No alteration in ploidy was seen in three separate clones of primary MEFs from *Fancd2*^−/−^ mice exposed to MMC (Fig. 2B). Therefore, cells from Fan1^nd/nd^ mice, but not *Fancd2*^−/−^ mice, show increased ploidy in a subset of cells in response to ICL induction. This suggests that defective ICL repair may be the cause of the karyomegaly aspect of KIN.

### Divergence between Fan1 and the FA pathway in ICL repair

**Figure 3.**
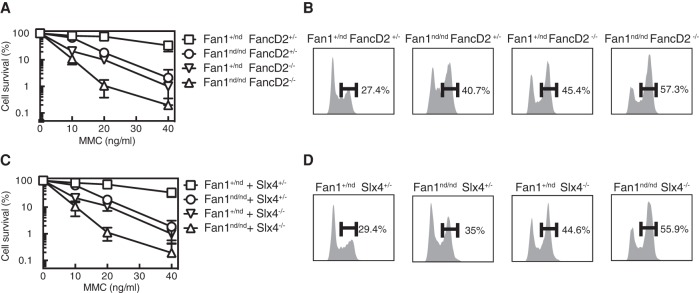
Fan1 is not epistatic to the FA pathway with respect to ICL repair. (*A*,*C*) Clonogenic survival analysis of MEFs of the genotypes indicated after exposure to an 18-h pulse of MMC at the concentration indicated. For each genotype, cell viability of untreated cells is defined as 100%. Data are represented as mean ± SD. *n* = 3. (*B*,*D*) MEFs of the genotypes indicated were exposed to MMC for 18 h before cells were fixed, stained with propidium iodide, and subjected to FACS analysis.

It has been shown previously that cells deficient in both Fan1 and components of the FA pathway are more sensitive to ICL-inducing agents than the respective single mutants (Yoshikiyo et al. 2010; Zhou et al. 2012). This finding suggested that the role of Fan1 in ICL repair is different from the FA pathway. We carried out similar analysis with our *Fan1*^nd/nd^ mice by crossing them with *Fancd2*^−/−^ mice to generate double mutants. MEFs from the *Fan1*^nd/nd^ *Fancd2*^−/−^ mice were more sensitive to MMC than the respective single mutants (Fig. 3A). Furthermore, whereas the severity of G_2_ arrest induced by MMC was similar in single-mutant MEFs, the strength of G_2_ arrest was substantially higher in double-mutant MEFs (Fig. 3B). To test further the notion that Fan1 and the FA pathway differ in their roles in ICL repair, we focused on *Slx4*, an FA gene that acts downstream from Fancd2 (Kim et al. 2011; Stoepker et al. 2011). Slx4 is a scaffold protein that binds to and regulates three separate structure-selective nucleases, all involved in ICL repair: Xpf–Ercc1, Mus81–Eme1, and Slx1 (Fekairi et al. 2009; Munoz et al. 2009; Svendsen et al. 2009; Castor et al. 2013). *Fan1*^nd/nd^ mice were crossed with *Slx4*^−/−^ mice, and MEFs from the *Fan1*^nd/nd^ *Slx4*^−/−^ double mutant showed increased sensitivity to MMC compared with the single mutants (Fig. 3C). Furthermore, although the severity of G_2_ arrest induced by MMC was similar in single-mutant MEFs, the strength of arrest was substantially higher in the *Fan1*^nd/nd^ *Slx4*^−/−^ double mutants (Fig. 3D). None of the mutant MEFs described above showed alteration in cell cycle distribution or G_2_ arrest prior to exposure to ICL-inducing agents (Supplemental Fig. S4A,B). These data confirm that Fan1 is not epistatic to the FA pathway with respect to ICL repair, suggesting that the two differ in the roles that they play in repairing ICLs.

### Genetic interaction between Fan1 and other ICL repair nucleases

**Figure 4.**
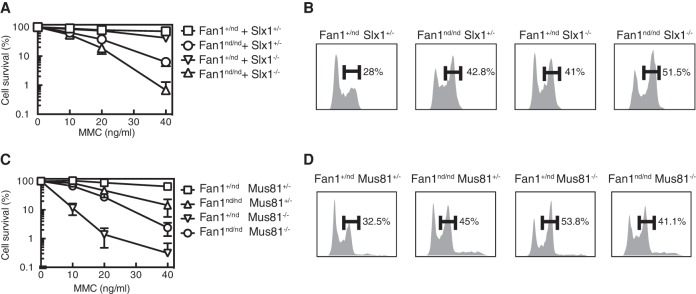
Genetic interaction between Fan1 and other ICL repair nucleases. (*A*,*C*) Clonogenic survival analysis of MEFs of the genotypes indicated after exposure to an 18-h pulse of MMC at the concentrations indicated. For each genotype, cell viability of untreated cells is defined as 100%. Data are represented as mean ± SD. *n* = 3. (*B*,*D*) MEFs of the genotypes indicated were exposed to MMC for 18 h before cells were fixed, stained with propidium iodide, and subjected to FACS analysis.

Next, we investigated functional relationships between Fan1 and other nucleases involved in ICL repair. To this end, we crossed *Fan1*^nd/nd^ mice with mice lacking Slx1, a nuclease involved in both ICL repair and Holliday junction resolution (Castor et al. 2013). MEFs from the *Slx1*^−/−^ mice show weak hypersensitivity to MMC, but, for the purposes of epistasis analysis, we used MMC concentrations at which the MMC sensitivity of *Slx1*^−/−^ MEFs was indistinguishable from wild-type MEFs. Using these conditions, we found that MEFs from *Fan1*^nd/nd^ *Slx1*^−/−^ mice were more sensitive to MMC than *Fan1*^nd/nd^ mutant MEFs (Fig. 4A), and the strength of G_2_ arrest was also substantially higher than in the respective single mutants (Fig. 4B). We also crossed *Fan1*^nd/nd^ mice with *Mus81*^−/−^ mice lacking the Mus81 subunit of the Mus81–Eme1 nuclease implicated in ICL repair and resolution of Holliday junctions (Ciccia et al. 2008). *Mus81*^−/−^ MEFs were more sensitive to MMC than *Fan1*^nd/nd^ MEFs, and *Fan1*^nd/nd^ *Mus81*^−/−^ MEFs were not more sensitive to MMC than the *Mus81*^−/−^ MEFs; if anything, the double-mutant MEFs appeared to be slightly less sensitive to MMC than *Mus81*^−/−^ cells (Fig. 4C). Consistent with these data, the strength of G_2_ arrest in the double mutants was not higher than in the single mutant with the strongest arrest (*Mus81*^−/−^); if anything, the arrest seen in the *Fan1*^nd/nd^ *Mus81*^−/−^ double-mutant cells was slightly weaker than in *Mus81*^−/−^ cells (Fig. 4D). None of the single- or double-mutant MEFs described above showed alteration in cell cycle distribution or G_2_ arrest prior to exposure to ICL-inducing agents (Supplemental Fig. S4C,D). Taken together, the data in this section show that Fan1 is not epistatic to Slx1–Slx4 in the repair of MMC-induced ICLs and that Fan1 may cooperate with Mus81–Eme1 in making incisions required for ICL repair.

In this study, we found that cells from mice lacking Fan1 nuclease activity show hallmarks of defective ICL repair, and the mice recapitulate hallmarks of KIN. The KIN symptoms in these mice are milder than in human KIN patients in that major defects in kidney function were not observed in the mice (data not shown). It is not yet clear why Fan1 inactivation affects kidneys in particular, but these organs filter blood and transport toxic waste chemicals, which may induce ICLs. Polyploidy and KIN have been linked to high levels of environmental toxins, such as ochratoxin A, and are also seen after administration of high doses of chemotherapeutic agents that induce ICLs (Godin et al. 1996). It is interesting to note that polyploidization (and renal failure) is observed in organisms with mutations in other ICL repair genes. For example, humans and mice defective in the DNA repair nuclease Xpf–Ercc1 show extreme polyploidization in the liver and kidney and have renal insufficiency (McWhir et al. 1993; Selfridge et al. 2001; Niedernhofer et al. 2006). This observation is consistent with polyploidy stemming from defective ICL repair. Similarly, we found that exposure to ICL-inducing agents can induce polyploidy in primary MEFs from *Fan1*^nd/nd^ mice. DNA content measurements indicate whole-genome or near-whole-genome doublings in the karyomegalic nuclei of Fan1-defective mice, which suggest that DNA rereplication or mitotic defects might be responsible for polyploidy. However, the underlying molecular mechanisms are unclear, and it will be interesting to investigate exactly how Fan1 prevents polyploidy.

If karyomegaly and, perhaps, the other symptoms of KIN are caused by defective ICL repair, then why do *Fancd2*^−/−^ mice or *Slx4*^−/−^ mice not show signs of KIN? Our observation that Fancd2-null cells do not become polyploid after a pulse of MMC provides a potential reason for why FA pathway mutations do not lead to KIN or at least the karyomegaly aspect of this disease. Another puzzling question is why humans with Fan1 mutations do not develop FA, especially given that Fan1-defective cells are very similar to cells from FA patients in showing the same hallmarks of defective ICL repair. The answers to this question may be inextricably linked to our findings that Fan1 is not epistatic to the FA factors Fancd2 and Slx4 in mice, adding to a growing body of work demonstrating that the role of Fan1 in ICL repair is not equivalent to the FA pathway. The basis of a functional difference between Fan1 and the FA pathway in ICL repair will be an important area of investigation. It is possible that Fan1 may respond to a subset of ICLs that is somehow different in nature from the ICLs dealt with by the FA pathway. This might reflect a difference in the chemical nature of the ICLs or a difference in the genomic context of the ICLs recognized by the two pathways.

There has been speculation that Fan1 may act in a manner redundant with other nucleases, particularly Slx1 (Klein Douwel et al. 2014; Zhang and Walter 2014). In this light, we found that Fan1 is not epistatic to Slx1 (Fig. 4). Slx1 can cleave a range of branched structures, including 5′ flaps that are also cleaved efficiently by Fan1 (Fekairi et al. 2009; Munoz et al. 2009; Svendsen et al. 2009). All of the Slx1 in cells is bound to Slx4, and Slx1 requires Slx4 for stability and activity (Munoz et al. 2009; Stoepker et al. 2011), so it is not surprising that Slx1 is similar to Slx4 in that neither factor is epistatic to Fan1 in ICL repair. In contrast, combining the Fan1-nd mutation with the Mus81 deletion does not lead to increased MMC sensitivity or enhanced G_2_ arrest. If anything, it appears that Fan1 mutation slightly weakens the MMC hypersensitivity and MMC-induced G_2_ arrest seen in *Mus81*^−/−^ cells. The basis for this observation is not yet clear. It may be that Fan1 and Mus81–Eme1 cooperate under normal circumstances in making incisions required for ICL repair, but, in the absence of Mus81–Eme1, Fan1 might inappropriately cleave intermediates of ICL repair that it cannot normally access. More work will be required to understand the functional relationships between Fan1 and Mus81–Eme1 and other nucleases involved in ICL repair at the mechanistic level, an effort that would be greatly facilitated by the development of better tools to probe the DNA transactions occurring during ICL repair in intact mammalian cells.

## Materials and methods

### Renal pathology

Animals were euthanized by means of carbon dioxide asphyxiation, and a complete post-mortem evaluation and dissection were performed. Kidneys were collected and fixed in 10% neutral buffered formalin for 48 h. Tissues were trimmed and processed to paraffin blocks. Three-micrometer-thick sections were stained with hematoxylin and eosin (HE) and evaluated in a genotype-blind manner. Karyomegaly in renal tubular epithelial cells was counted in 40 high-power fields (HPFs) at 400× magnification in the HE-stained sections of the kidney and liver. Karyomegaly was counted when the size of the nucleus in a renal tubular epithelial cell exceeded three times the size of the nucleus of other tubular epithelial cells within the same microscopic field.

### Ploidy analysis of karyomegalic nuclei from renal tubular epithelium

DNA ploidy analysis was performed on nuclei separated from paraffin-embedded tissue and stained with Feulgen periodic acid–Schiff reagent on monolayers as described previously (Sperandio et al. 2013). Images were captured and analyzed on Ploidy Work Station software (Room4 Group Ltd.), which automates image capture based on a Zeiss Axioplan II microscope and Axiocam MRm camera (Room4 Group Ltd.), with original magnification 40× producing images at162 nm/pixel. Integrated optical density, proportional to individual nuclear DNA content, was plotted against nuclear number after incomplete or overlapping profiles were excluded.

### Phospho-H2AX immunohistochemistry

Automated immunohistochemistry was performed with the DAKO Autostainer (DAKO). Briefly, following dewaxing, rehydration, quenching of endogenous peroxidase with 3% H_2_O_2_ in PBS, and heat-induced epitope retrieval (citrate buffer at pH 6.0 in a pressure cooker), sections of kidneys were incubated with a rabbit monoclonal antibody directed against phosphorylated histone H2AX (Cell Signaling Technology, 9718) used at 1:400 dilution. Visualization was performed with Envision anti-rabbit (DAKO) followed by development of the colorimetric reaction with DAB (DAKO) as a chromogen. Tubular epithelial cells with positive nuclear staining for phospho-H2AX were counted in 40 HPFs at 400× magnification.

### Isolation and immortalization of MEFs

Embryos (13.5 d post-coitum) were derived from timed matings between heterozygous parents. After decapitation, the heads were used for genotyping. The red organs were removed, and the embryo was minced and resuspended in 1 mL of trypsin and incubated for 15 min at 37°C before the addition of 10 mL of growth medium. Cells were plated and allowed to attach overnight before cells were washed with fresh medium to remove debris. When cells reached confluency, they were split and replated, and this was considered passage 1. All MEFs, except where stated, were immortalized using SV40 large T antigen.

### Cell culture

All cells were kept at 37°C under humidified conditions with 5% CO_2_. 293T cells were grown in DMEM, 10% FBS, L-glutamine, and penicillin/streptomycin. For MEFs, medium was supplemented with Na-pyruvate and nonessential amino acids.

### Detection of G_2_ arrest by flow cytometry

Low-passage, low-confluence cultures of asynchronously growing MEFs were treated with 50 ng/mL MMC (Sigma-Aldrich) or 10 ng/mL DEB for 48 h. Following trypsinization, cells were collected by centrifugation, washed in PBS, and fixed in ice-cold 70% ethanol. Cells were centrifuged, washed in PBS, and treated for 15 min at 37°C with 50 μg/mL RNase A. Cells were stained at room temperature with propidium iodide (PI) solution (2 mM MgCl_2_, 10 mM PIPES buffer, 0.1 M NaCl, 0.1% Triton X-100, 0.01 mg/mL PI) and analyzed on a FACS Canto flow cytometer (BD Biosciences). Cell cycle profiles were created using FlowJo analysis software (Tree Star, Inc.).

### Clonogenic survival analysis

MEFs were seeded in triplicates in 10-cm dishes and allowed to attach before treatment. Genotoxin was added to cells for 24 h before medium was replaced with fresh growth medium. After 10 d, cells were washed, fixed, and stained with Giemsa. The number of colonies with >100 cells was counted. For each genotype, cell viability of untreated cells was defined as 100%. Data are represented as mean ± SD from three independent experiments.

All cDNA plasmids, antibodies, and recombinant proteins generated for this study are available on request through our reagents Web site (https://mrcppureagents.dundee.ac.uk). Full details of all other Materials and Methods are in the Supplemental Material.

## Supplementary Material

Supplemental Material
