# Supplementary material for: Karyomegalic interstitial nephritis and DNA damage-induced polyploidy in Fan1 nuclease-defective knock-in mice
Source: Genes Dev. 2016 Mar 15;30(6):639–44. doi: 10.1101/gad.276287.115 (PMC4803050; doi:10.1101/gad.276287.115)
Supplement: Supplemental Material [file supp_30_6_639__index.html]

Supplemental Material 

# Karyomegalic interstitial nephritis and DNA damage-induced polyploidy in Fan1 nuclease-defective knock-in mice

## Supplemental Material

**Files in this Data Supplement:**

- Supp Material.pdf
