# Supplementary material for: Karyomegalic interstitial nephritis and DNA damage-induced polyploidy in Fan1 nuclease-defective knock-in mice
Source: Genes Dev. 2016 Mar 15;30(6):639–44. doi: 10.1101/gad.276287.115 (PMC4803050; doi:10.1101/gad.276287.115)
Supplement: Supplemental Material [file supp_30.6.639_SuppMaterial.pdf]

Supplemental Information

This file contains:

Figs S1-4

Fig. S1 The Fan1-nd mutant is devoid of nuclease activity

Fig. S2 Cells from $Fan1^{nd/nd}$ mice show hallmarks of defective ICL repair

Fig. S3 $Fan1^{nd/nd}$ mice show altered ploidy

Fig. S4 Normal cell cycle profiles in $Fan1^{nd/nd}$, $Slx4^{-/-}$, $Slx1^{-/-}$ and $Mus81^{-/-}$ MEFs

Table S1-2

Table S1 Mendelian ratios of Fan1-nd mice

Table S2 Scoring of kidney phenotypes in WT and Fan1-nd mice

Supplementary Figure Legends

Fig. S1. The Fan1-nd mutant is devoid of nuclease activity

$Fan1^{-/-}$ U2OS cells were transfected with plasmids expressing Fan1 or Fan1-nd with a N-terminal FLAG epitope tag, or a control plasmid with FLAG only. Anti-FLAG immunoprecipitate were incubated with a synthetic FITC-labelled 5' flap as described in Supplemental Methods. Reaction products were subjected to denaturing PAGE (top panel). FLAG-FAN1 levels in the Immunoprecipitates where analysed by SDS-PAGE followed by western blotting (bottom panel).

Fig. S2. Cells from $Fan1^{nd/nd}$ mice show hallmarks of defective ICL repair

A. MEFs of the genotypes indicated were exposed to MMC (50 ng/ml) or DEB (10 ng/ml) for 48 h before cells were fixed, stained with propidium iodide and subjected to FACS analysis. **B.** Metaphase spreads of MEFs treated with MMC (50 ng/ml) or DEB (10 ng/ml) for 24 h were stained with DAPI and analysed for the presence of radial and broken chromosomes. 20 metaphase spreads in 2 independent experiments were analysed for each cell line either untreated or treated with MMC and the number of abnormalities per metaphase was determined.

Fig. S3. Altered ploidy in kidneys from $Fan1^{nd/nd}$ mice

DNA ploidy histograms for kidney from $Fan1^{+/nd}$ and $Fan1^{nd/nd}$ mice.

Fig. S4. Normal cell cycle profiles in $Fan1^{nd/nd}$, $Slx4^{-/-}$, $Slx1^{-/-}$ and $Mus81^{-/-}$ MEFs

MEFs of the genotypes indicated were fixed, stained with propidium iodide and subjected to FACS analysis.

Supplemental materials and methods

Mouse strains and husbandry

Animals were housed under specific pathogen free conditions in accordance with UK and EU regulations. All procedures were carried out in accordance with University of Dundee and United Kingdom Home Office regulations. Generation of Fan1$^{nd/nd}$ mice was described previously (Lachaud et al., 2016). Fancd2$^{-/-}$ mice, also described previously, were a kind gift from Alan d'Andrea (Parmar et al., 2010). All mice were backcrossed 4 times before analysis. Slx1$^{-/-}$ and Slx4$^{-/-}$ mice were described previously (Castor et al., 2013).

Antibodies

Antibodies against mouse Fan1 were raised in sheep against the 300 first amino acid of mouse Fan1 fused to GST. Antibodies were affinity purified from serum from sheep S778D using immobilized antigen, and used at 0.5 μg/ml for 1h for western blotting. Anti-GAPDH (14C10) antibodies were purchased from Cell Signalling. All secondary antibodies were purchased from Invitrogen. An anti-GFP antibody was purchased from Abcam. Both Anti-GAPDH and Anti-GFP were used according to the manufactures instructions.

DNA constructs

The full-length coding regions for the relevant human proteins were generated by PCR using IMAGE consortium EST clones. Full-length coding regions for the relevant mouse proteins were generated by RT-PCR (Takara PrimeScript High Fidelity RT-PCR Kit). Point mutations were introduced by quickchange mutagenesis. The sequence integrity was confirmed by sequencing analysis. mouse cDNA were cloned into pcDNA or pBabe.puro vectors.

Measurment of nuclease acitivity in FLAG-FAN1 immunoprecipitates

Cells were lysed in ice–cold RIPA buffer: (50 mM Tris-HCl pH 7.5, 150 mM NaCl, 1% NP40, 1% Na-deoxycholate, 2 mM EDTA, 0.1% SDS) supplemented with protease inhibitors (Roche) and 50 U/ml of benzonase (Novagen). Lysates were

pre-cleared with protein G sepharose beads for 30 min at 4°C. All immunoprecipitations were carried out for 1h at 4°C using 20 µl of FLAG-M2 agarose beads (Sigma) per 5 mg of whole cell extract. The oligonucleotides used to assemble the synthetic 5’ flap substrate had the following sequences (5’ to 3’).

Oligos were annealed by heating to 90°C for 10 min followed by slow-cooling for 30 min. The 5’ flap substrate was then purified by native PAGE, eluted using he crush and soak method, and then ethanol-precipitated. Beads were pre-incubated for at least 5 min with DNA substrates (5 nM) at 37°C in 25 mM Tris-HCl (pH 7.5), 10 mM NaCl,15 mM KCl, and 0.1 mg/ml BSA to allow binding to occur. The reaction was started by the addition of 1 mM MnCl2 and stopped by the addition of 2 mM EDTA. The samples were then boiled at 95°C for 10 min and analyzed by denaturing PAGE (15% polyacrylamide and 8 M urea).

Reference

Castor, D., Nair, N., Declais, A.C., Lachaud, C., Toth, R., Macartney, T.J., Lilley, D.M., Arthur, J.S., and Rouse, J. (2013). Cooperative Control of Holliday Junction Resolution and DNA Repair by the SLX1 and MUS81-EME1 Nucleases. Mol Cell.

Lachaud, C., Moreno, A., Marchesi, F., Toth, R., Blow, J.J., and Rouse, J. (2016). Ubiquitinated Fancd2 recruits Fan1 to stalled replication forks to prevent genome instability. Science.

Parmar, K., Kim, J., Sykes, S.M., Shimamura, A., Stuckert, P., Zhu, K., Hamilton, A., Deloach, M.K., Kutok, J.L., Akashi, K., et al. (2010). Hematopoietic stem cell defects in mice with deficiency of Fancd2 or Usp1. Stem Cells 28, 1186-1195.

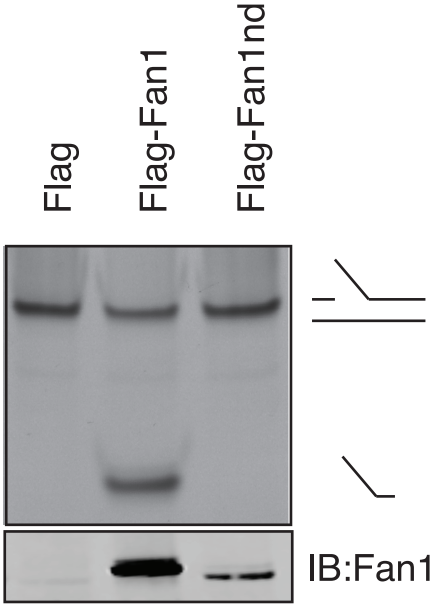
Flag
Flag-Fan1
Flag-Fan1nd
IB:Fan1

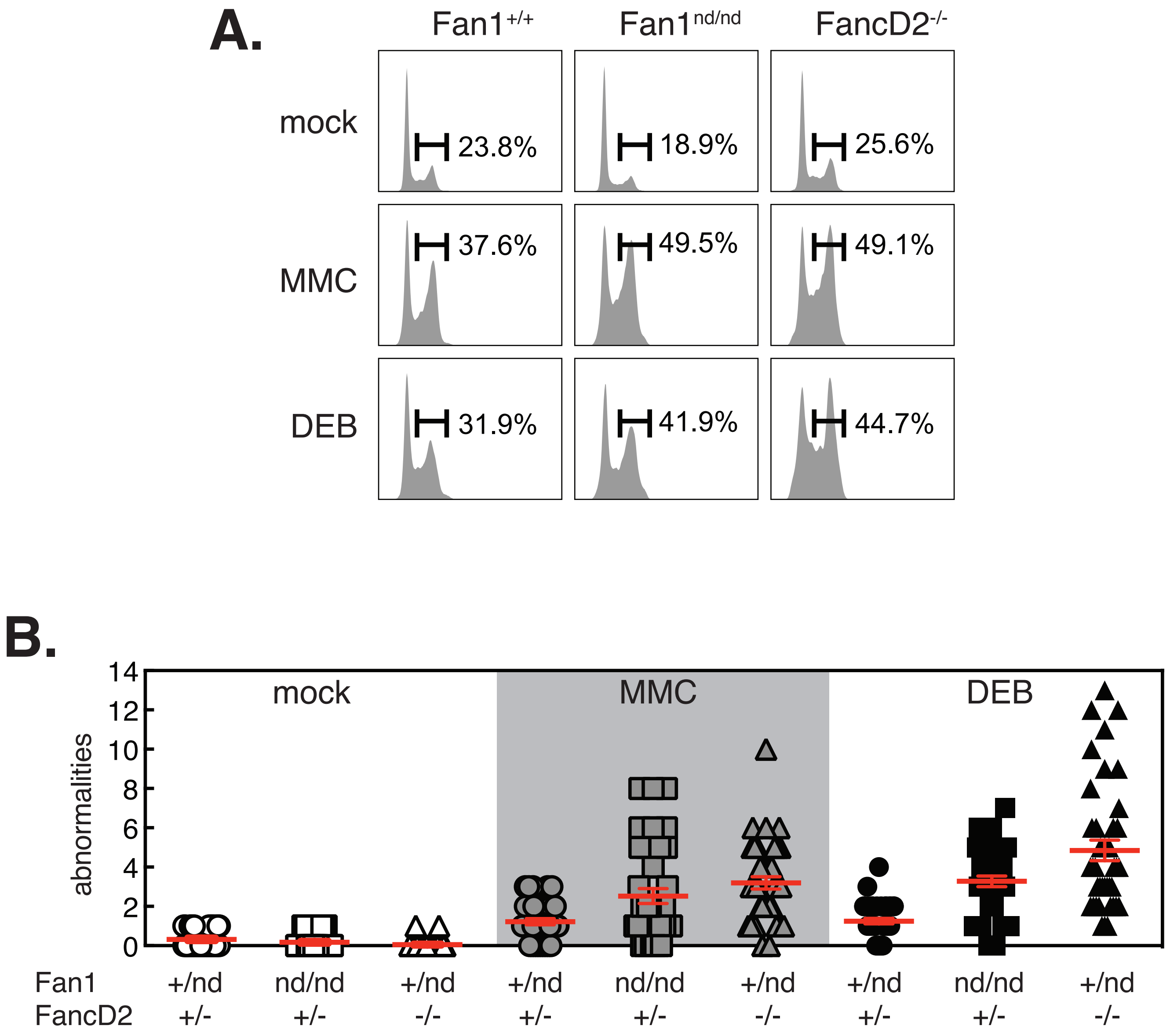
A.
Fan1+/+
Fan1nd/nd
FancD2-/-
mock
MMC
DEB
23.8%
18.9%
25.6%
37.6%
49.5%
49.1%
31.9%
41.9%
44.7%
B.
abnormalities
0
2
4
6
8
10
12
14
mock
MMC
DEB
Fan1
FancD2
+/nd
+/-
nd/nd
+/-
+/nd
-/-
+/nd
+/-
nd/nd
+/-
+/nd
-/-
+/nd
+/-
nd/nd
+/-
+/nd
-/-

Lachaud_Fig.S3

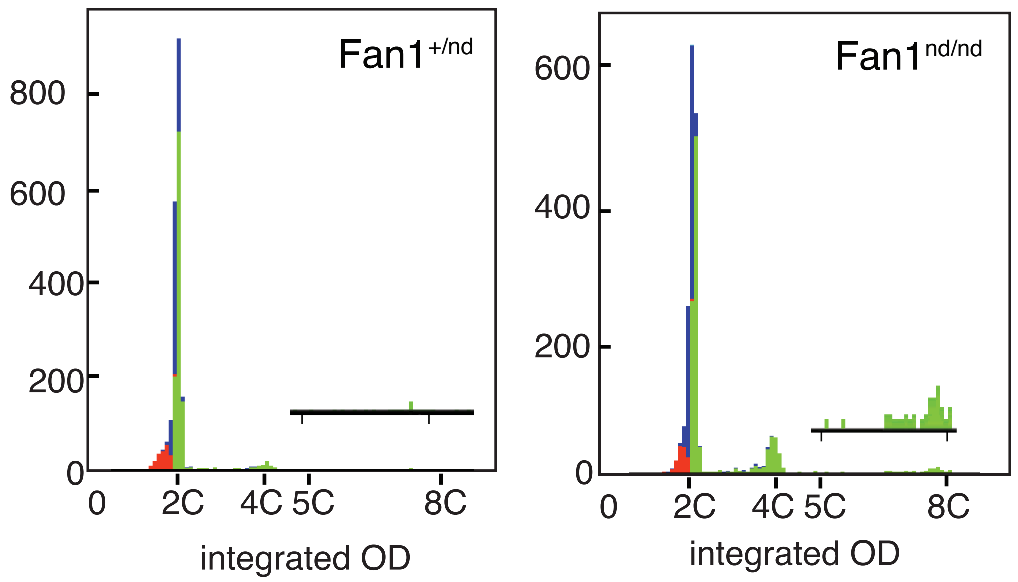

Lachaud_Fig.S4

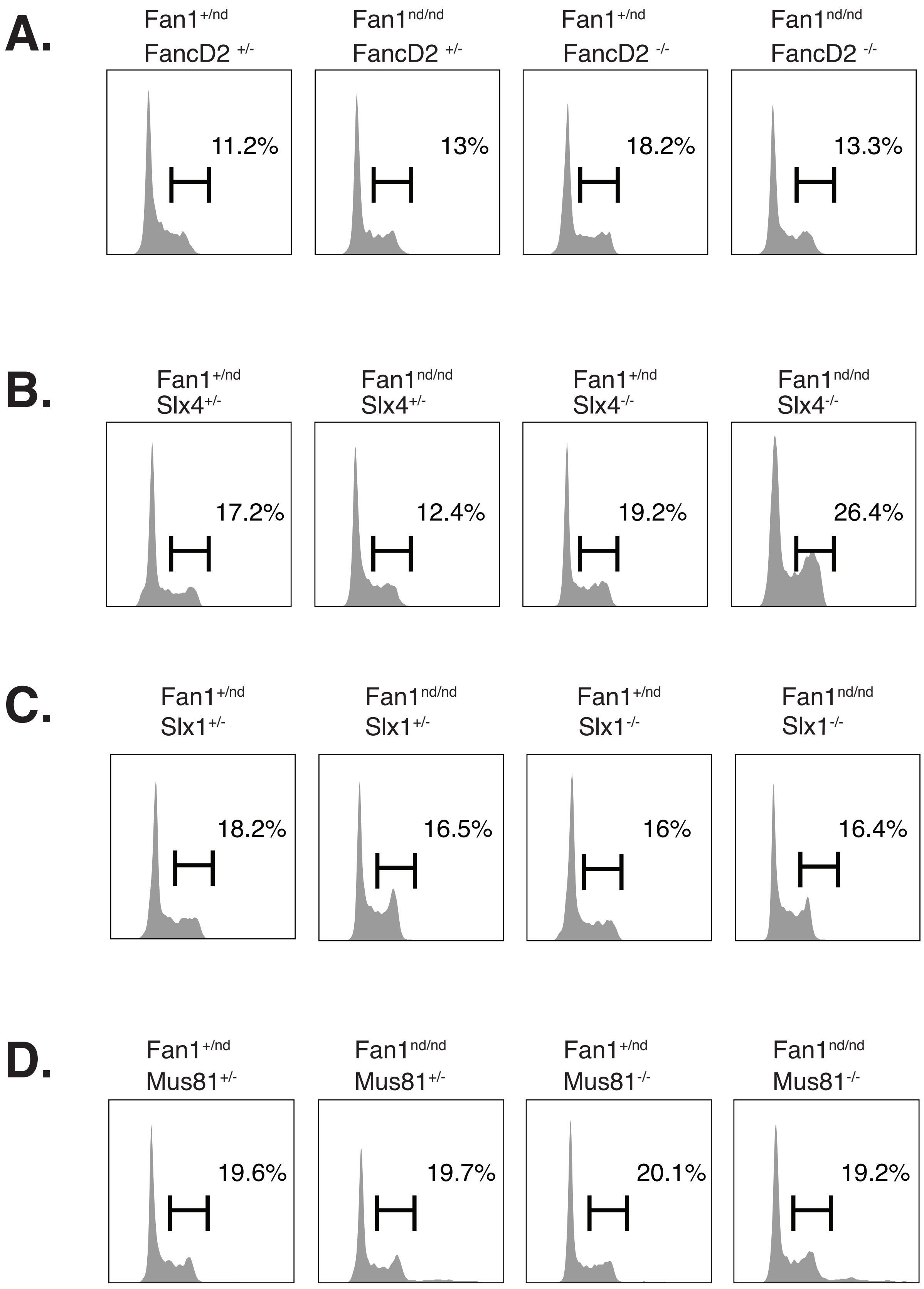

Lachaud_ Table S1

P:	Fan1 $^{nd/+}$ x Fan1$^{nd/+}$		
F1	Fan1$^{+/+}$	Fan1$^{nd/+}$	Fan1$^{nd/nd}$
expected	25%	50%	25%
day 21 (n=113)	22.10%	51.30%	26.50%

Chi-sqaure test
Two-tailed P values

0.8701

Lachaud_ Table S2

Mouse ID (gender)	Age (days)	Genotype	Tubular karyomegaly #	Tubular dilation /degeneration/atrophy #	Inflammatory cell infiltration #
153793 (F)	328	hom	++	+	++
155710 (F)	328	het	-	+	-
160063 (F)	286	het	-	-	+
165964 (F)	257	hom	++	+	++
155701 (M)	328	hom	+++	-	+++
152756 (M)	334	het	-	+	+
189831 (F)	176	hom	+	+	-
189832 (F)	176	hom	++	+	-
197000 (F)	141	wt	-	++	+
191016 (M)	170	wt	-	-	+
191017 (M)	170	wt	-	-	-
191018 (M)	170	wt	-	++	++
189640 (M)	180	hom	++	++	-
189643 (M)	180	hom	++	-	-
184598 (F)	587	het	-	-	++
191022 (F)	542	het	-	+	++
184207 (F)	589	hom	+++	+	+
184041 (M)	590	hom	++++	-	+
184043 (M)	590	het	+	-	+
189829 (M)	548	het	-	-	++
184201 (M)	589	hom	++++	+++	+++
187665 (M)	565	hom	+++	+	-
184046 (M)	590	wt	-	+	+
189645 (M)	552	wt	-	-	+
189642 (M)	552	wt	-	-	+
189833 (F)	582	hom	+++	++	+++
184599 (F)	621	hom	+++	+	++
184597 (F)	621	het	-	-	+
184595 (F)	621	het	-	-	+
184594 (F)	621	het	-	+	+
185936 (M)	612	hom	++++	+++	+++
189644 (M)	586	het	-	-	+
189641 (M)	586	het	+	-	+

microscopic changes are reported as

- = absent

+ = minimal

++ = mild

+++ = moderate

++++ = marked

NS = not specified
